# The association between Ki-67 expression and the clinical pathological characteristics of colorectal cancer: A protocol for a systematic review and meta-analysis

**DOI:** 10.1097/MD.0000000000019996

**Published:** 2020-05-22

**Authors:** Jing Li, Zhi-ye Liu, Hai-bo Yu, Xiu-sheng Qu, Qing Xue, Hai-tao Yu, Christina Weeks

**Affiliations:** a Department of Physiology, Jiamusi University, School of Basic Medical Sciences; b Department of Radiotherapy and Chemotherapy, First Affiliated Hospital of Jiamusi University; c Department of Cardiology, First Affiliated Hospital of Jiamusi University; d 7th Class of Clinical Medicine of 2016 Grade, Jiamusi University; e Department of Anorectal, First Affiliated Hospital of Jiamusi University, Jiamusi, 154002, China; f Medical School, University of Edinburgh, Edinburgh, EH8 9AG, UK.

**Keywords:** clinical pathological characteristic, colorectal cancer, Ki-67 expression

## Abstract

**Background:**

This study will explore the association between Ki-67 expression and clinical pathological characteristics (CPC) of colorectal cancer (CC).

**Methods:**

We will search relevant studies from electronic databases (Cochrane Library, PUBMED, EMBASE, Scopus, Cumulative Index to Nursing and Allied Health Literature, China Biology Medicine, and China National Knowledge Infrastructure) from beginning to April 1, 2020 without language and publication time limitations. We will consider all case-controlled studies (CCSs) or randomized controlled studies (RCSs) investigating the association between Ki-67 expression and CPC of CC. We will appraise study quality of CCSs by Newcastle–Ottawa Scale, and RCSs by Cochrane risk of bias tool. Statistical analysis will be carried out by Review Manager 5.3 software.

**Results:**

The present study will explore the association between Ki-67 expression and CPC of CC.

**Conclusion:**

Its findings may summarize scientific evidence of the association between Ki-67 expression and CPC of CC, and may provide helpful evidence for clinical practice.

Systematic review registration: PROSPERO CRD42020173795.

## 1 Introduction

Colorectal cancer (CC), also known as colorectal adenocarcinoma, is the third most commonly diagnosed cancer in males and the second in females.^[1–4]^ It is also one of the most leading reasons of cancer mortality around the world.^[5–8]^ It has been reported that about 1,096,000 new cases of colon cancer and about 704,000 new cases of rectal cancer were diagnosed in 2018.^[3]^ Types of CC include colorectal adenocarcinoma, gastrointestinal carcinoid tumors, primary colorectal lymphomas, gastrointestinal stromal tumors, leiomyosarcomas, and melanomas.^[9–14]^

Previous studies have reported the association between Ki-67 expression and clinical pathological characteristics (CPC) in patients with CC.^[15–31]^ However, no study has explored the association between Ki-67 expression and CPC in patients with CC at evidenced-medicine level. Thus, this study firstly investigated the association between Ki-67 expression and CPC in patients with CC.

## 2 Methods

### 2.1 Study registration

This study has been registered on PROSPERO (CRD42020173795), and has been reported according to the Preferred Reporting Items for Systematic Reviews and Meta-Analysis (PRISRMA) Protocol statement guidelines.^[32]^

### 2.2 Eligibility criteria

#### 2.2.1 Types of trials

All potential case-controlled studies (CCSs) or randomized controlled studies (RCSs) that explored the association between Ki-67 expression and CPC of CC will be included. We will exclude any other studies, such as review, comment, and uncontrolled studies.

#### 2.2.2 Types of subjects

We will include all patients who were diagnosed as CC, regarding gender, age, and race.

#### 2.2.3 Types of exposures

In the experimental group, CC tissues from patients with CC were collected.

In the control group, normal tissues adjacent to the CC in patients with CC were harvested.

#### 2.2.4 Types of outcome measurements

Primary outcomes are protein and gene expressions of Ki-67. Gene expression of Ki-67 was measured by real-time polymerase chain reaction. Protein expression of Ki-67 was detected by immunofluorescence or western blot test.

Secondary outcomes are associations between Ki-67 expression and gender, age, clinical stages, tumor sites, pathological features, and lymph node metastasis.

### 2.3 Literature search and search strategy

The primary sources of literature search will be sought in electronic databases (Cochrane Library, PUBMED, EMBASE, Scopus, Cumulative Index to Nursing and Allied Health Literature, China Biology Medicine, and China National Knowledge Infrastructure) from initiation to April 1, 2020 with no language and publication time restrictions. The CCSs or RCSs that explore the association between Ki-67 expression and CPC of CC will be included. The search strategy details of Cochrane Library are summarized (Table 1). Similar search strategies will be adapted to other electronic databases.

**Table 1:**
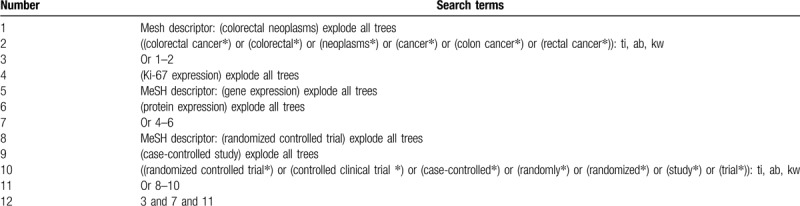
Search strategy used in Cochrane Library database.

The secondary sources are Google Scholar, conference abstracts, clinical registrations, and reference lists of relevant reviews.

### 2.4 Study selection

Two researchers will independently identify titles/abstracts of all retrieved studies to remove all irrelevant records. Full-papers of potential studies will be read carefully against eligibility criteria. Any uncertainties between 2 researchers will be solved by a third researcher through discussion. We will record reasons for all excluded literatures. The procedure of study selection will be exerted in a PRISRMA flow chart.

### 2.5 Data extraction and management

Two researchers will independently extract data using a predefined data extraction sheet. The extracted information comprises of general information (e.g., first author, year of publication, region, gender, ethnicity, disease types, and diagnostic criteria), types of studies (CCSs or RCSs), study quality, outcome measurements (primary and secondary outcomes, and any others), results, and findings. Any differences between 2 researchers will be resolved by a third researcher through discussion.

### 2.6 Study quality assessment

Two researchers will independently assess study quality for all eligible studies. We will resolve any differences with the help of a third researcher. We will evaluate study quality for CCSs by Newcastle–Ottawa Scale, and that for RCSs by Cochrane risk of bias tool.

### 2.7 Statistical analysis

RevMan 5.3 software will be used for statistical analysis. We will estimate the treatment effects of dichotomous data as risk ratio and 95% confidence intervals (CIs), and will calculate the treatment effects of continuous data as mean difference or standardized mean difference and 95% CIs. We will employ *I*^2^ test to explore statistical heterogeneity across eligible studies. *I*^2^* *≤ 50% indicates homogeneity, and a fixed-effects model will be used to pool data. A meta-analysis will be performed if it is necessary. *I*^2^ > 50% exerts considerable heterogeneity, and a random-effects model will be utilized to synthesize data, and a subgroup analysis will be carried out to detect the sources of obvious heterogeneity. In addition, we will also report study results by a narrative summary.

### 2.8 Additional analysis

#### 2.8.1 Subgroup analysis

A subgroup analysis will be performed to identify heterogeneity sources based on the different types of studies, study characteristics, and outcome indicators.

#### 2.8.2 Sensitivity analysis

A sensitivity analysis will be carried out to test robustness of study findings by removing low quality studies.

#### 2.8.3 Reporting bias

Reporting bias will be examined by funnel plot and Egger's regression test if more than 10 studies are included.^[33,34]^

### 2.9 Dissemination and ethics

This study will not harvest individual patient data, thus, no ethical approval is required. We expect to publish this study on a peer-reviewed journal or a conference presentation.

## 3 Discussion

This study will explore the association between Ki-67 expression and CPC in patients with CC. We will collect potential studies from electronic databases and other literature sources. All possible studies will be included to investigate the association between Ki-67 expression and CPC in patients with CC. Although previous studies have reported that Ki-67 expression is associated with CPC of CC, their results are still inconsistent. This study will perform a comprehensive synthesis to assess the association between Ki-67 expression and CPC in patients with CC. We hope that this study can provide a helpful evidence of the association between Ki-67 expression and CPC of CC, which is beneficial to both patients and practitioners.

## Author contributions

**Conceptualization:** Jing Li, Hai-bo Yu, Qing Xue, Hai-tao Yu, Weeks Christina.

**Data curation:** Jing Li, Zhi-ye Liu, Hai-bo Yu, Xiu-sheng Qu, Qing Xue.

**Formal analysis:** Jing Li, Zhi-ye Liu, Xiu-sheng Qu, Qing Xue, Hai-tao Yu.

**Funding acquisition:** Jing Li.

**Investigation:** Qing Xue.

**Methodology:** Hai-bo Yu, Xiu-sheng Qu, Qing Xue, Hai-tao Yu.

**Resources:** Jing Li, Zhi-ye Liu, Hai-bo Yu, Xiu-sheng Qu, Qing Xue, Hai-tao Yu.

**Software:** Zhi-ye Liu, Xiu-sheng Qu, Qing Xue, Hai-tao Yu.

**Validation:** Jing Li, Zhi-ye Liu, Hai-bo Yu, Xiu-sheng Qu, Hai-tao Yu, Weeks Christina.

**Visualization:** Hai-bo Yu, Xiu-sheng Qu, Qing Xue, Hai-tao Yu, Weeks Christina.

**Writing – original draft:** Jing Li, Zhi-ye Liu, Hai-bo Yu, Xiu-sheng Qu, Qing Xue, Hai-tao Yu, Weeks Christina.

**Writing – review & editing:** Jing Li, Zhi-ye Liu, Xiu-sheng Qu, Qing Xue, Hai-tao Yu, Weeks Christina.
